# Cancer Viewed and Treated from the Standpoint of the General Practitioner, with Reports of Cases

**Published:** 1898-11

**Authors:** Bittle C. Keister

**Affiliations:** South Boston, Va.


### CANCER VIEWED AND TREATED FROM THE STANDPOINT OF THE GENERAL PRACTITIONER, WITH REPORTS OF CASES.

By BITTLE C. KEISTER, A.M., M.D.,
SOUTH BOSTON, VA.

The etiology of cancer is as yet very obscure, hence I shall not attempt any elaborate elucidation of a subject that is now agitating the minds of the scientific world.

Speculative authors tell us that it may be a parasitic disease, and, hence, may partake largely of the etiology of tuberculosis.

Various forms of bacteria have been observed from time to time in carcinoma by careful investigators. It is evident that these organisms form in cancer, and it is probable that they produce inflammations and necroses in the tumor, and in some cases, possibly, they have some connection with the cachexia.

Scheuerlen reported in 1887 a cancer bacillus which had been obtained by culture. The bacilli were short, and were capable of developing spores. These organisms, when inoculated into the mammary gland of dogs, produced tumors containing epithelial cells.

Professor Sanfelice, of the University of Cagliari, in Sardinia, and Professor Roncali, of the University Surgical Clinic in Rome, in 1895 published some very important data bearing on the etiology of cancer. Their remarkable experimental results with the blastomycetæ, as the active agents in the causation of cancer, has had much to do in producing a revolution of ideas on the pathology of this fell disease. By inoculation with these cultures, they have produced tumors in animals, which bear the strongest possible resemblance to those neoplasms from which the cultures were originally made. Without going so far as to say that this can be done in every instance, or that all cancers are necessarily of parasitic origin, one is justified by these results in at least maintaining that some cancers are positively of parasitic origin.

The presence of intracellular organisms of quite a different character from bacteria has created much speculation during the last few years. Since the anatomical nature of cancer is better understood, it is known that peculiar cell-like bodies are a characteristic feature of the disease. These bodies were supposed to be cells undergoing degenerative changes. Schutz thinks that most of the questionable intracellular structures found in carcinomata should be regarded as due to leucocytes, which have become embedded in the cell. Klebs, after careful study and experiment, decides that there are no positive grounds for regarding these cells as parasites. He sees, in the presence of these cells, within the epithelial cells, evidence apparently of the old French theory of the *action de présence*—the leucocytes exerting a fructifying influence upon the cancer-cells and causing them to multiply. Many still hold to the old idea that they are degenerated epithelial cells.

All attempts to cultivate these cells from cancer growth appear to have failed, and the number of cases in which cancer has been inoculated successfully into animals is exceedingly limited. Councilman does not consider these structures parasitic, having seen them in many other morbid processes as well as in cancer. He does not think the parasitic origin has yet been proven, and, on theoretical grounds, thinks it is hardly likely to be. Park, however, sees in these investigations sufficient to encourage the hope that surgeons are on the eve of great discoveries, which will settle the question of the origin of cancer.

Cancer is said to be less common in tropical than in temperate climates. Haviland proved the disease to be most prevalent in damp and in low-lying districts in England. It is said to be less frequently seen in Turkey, in Egypt, and in the West Indies. Negroes are generally supposed in America to be much less afflicted with cancer than the white race. In England statistics show that there are about 30,000 patients suffering at all times from cancer.

According to Park, the mortality of cancer is larger in and about western New York and the adjoining region than in any other part of the United States, save a limited area in California. Shattock has recently called attention to the fact that cancer-like tubercle may repeatedly show itself in certain houses. This author reported a series of four cases of cancer occurring within fourteen years, in persons unrelated by blood who were living in a single house.

Powers reports the history of three housekeepers who slept in succession for several years in the same house and the same bedroom. The first lived in the room for thirteen years and died of cancer of the stomach; the second, after a residence of twenty years, died of cancer of the liver; the third died at the end of eight years of cancer of the breast and uterus. They were all in good health previous to the time of their installment in the house.

I am of the opinion that cancer, like tuberculosis, where there is a family predisposition to the disease, requires only very slight cause to light up the disease. A slight blow on the breast may produce a nidus from which cancer may develop. This is equally true of other localities of the body, as the lip, the tongue, the larynx, and the mucous membrane of the nose, which, by being constantly irritated from external causes, such as chewing and smoking tobacco, the disease may show itself sooner or later.

Out of three hundred and three patients who were suffering from carcinoma of the lip, tongue, nose and pharynx (reported by Williams, Whitehead and Pennell), sixty per cent. used tobacco, and the majority smoked a pipe; and nineteen per cent. had had syphilis. Other local causes were direct injury in eleven cases, ulcers from bad teeth in thirty-seven, ichthyosis in fourteen, localized syphilis in fourteen, and glossitis in three—a total of seventy-nine cases out of one hundred and ninety-four, or forty per cent. of the cases (noted by Curtis) as due to local causes.

It would therefore appear that local irritation played a very important part in the etiology of the disease.

The most usual situations of carcinoma of the mouth are in the cheeks (where it often appears to be directly due to ulceration set up by rough teeth) and in the floor of the mouth. In both of these localities, the disease is rapidly fatal, and is apt to return after removal.

Carcinoma of the tongue is one of the most intractable forms of the disease, probably because of the constant movement of the organ, its liability to injury, and the great tendency of the mouth to sepsis. The general health is very soon affected, and the lymphatic channels are usually invaded early; in fact, Sachs cites instances in which they were involved as early as five weeks after the disease was noticed, although in some chronic cases the glands may escape for a long time. In fifty-two cases they were the only glands attacked, while five times the infection skipped them and appeared in the cervical, submental, or retromaxillary glands. This goes to prove the uncertain course that the disease may take; hence, we can very often be mistaken in locating the disease, and are tempted to remove important glands simply because it is customary to do so.

During the past five years I have tried to keep a record of my cancer cases, some of which I beg leave to report, as follows:

CASE 1.—A young lady, seventeen years old, whose grandmother died of carcinoma of the uterus at the age of forty-eight; one aunt and two uncles are victims of tuberculosis. This patient received a severe blow on her right cheek two years previous, from which she suffered intense pain for several months afterwards. Eighteen months after this she complained of a swelling of the parotid gland, which continued to grow worse until an abscess formed and was lanced by her family physician. The discharge continued several months, when she was brought to me for consultation. On examination I discovered several small sinuses in the neighborhood of the right parotid gland and cheek-bone, all of which were discharging a watery fluid, which was very offensive. The submaxillary gland on the right side was somewhat enlarged. The patient appeared to be in good health, except the sallow expression about the face.

I advised an immediate operation with the knife, but the father of the patient objected to the knife, and asked whether a milder form of treatment could not be substituted. I then suggested a thorough cauterization with the electrocautery, to which both the patient and father readily assented. After a careful preparation of the parts I decided to try caustic potash as a preliminary step, and followed this with electrocautery, which I did very carefully and thoroughly.

Previous to operating I gave the patient a hypodermic injection of one-third of a grain of sulph. morphia and a sixty-fourth of a grain of sulph. atropia. I also made a local application of equal parts of shaven ice and chloride sodium. The patient stood the operation well, and was conversing during the time of operating, and was able to return to her home, seven miles in the country, two hours later. I saw the patient ten days after this, and was delighted to find the wound healing very kindly by granulation. The only dressing that I prescribed was a twenty per cent. solution chloral hydrate in distilled water, directing the parts to be kept clean and moist with this lotion. The wound healed entirely in twenty days. The swelling in the submaxillary gland also subsided, and the patient has remained well up to the present writing, having reached the three-year limit.

CASE 2.—A man, thirty-eight (38) years old, married, and the father of seven living children; some tuberculosis in his father's family; his grandfather died of cancer of the face. The rest of his family history was uncertain. He stated that he had never suffered from any form of venereal disease; this statement, however, I doubted very much. He complained of soreness and pain about his genital organs, and stated that he had been operated upon about six months previously for constriction of the foreskin.

Examination of the penis revealed considerable swelling of the entire organ, with sloughing and offensive discharge, covering the glans-penis. The end of the penis was very large, measuring nine inches in circumference, and presented the appearance of a cauliflower. The meatus urinarius was almost entirely occluded, the urine dribbling continuously, causing the patient additional pain and worry. The discharge and sloughing of the glans-penis was so offensive that it was necessary to keep antiseptic dressings applied constantly.

On the 24th of April, 1897, with the assistance of Dr. T. W. Williamson, of Houston, in whose neighborhood the patient resided, I amputated the penis just one inch from the junction of the pubes.

In performing the operation I did not follow the usual method of making a complete circular incision, but, after putting the skin on the stretch, I made a sloping incision from above downwards and outwards through the corpora-cavernoso, and when I reached the corpus-spongiosum I allowed about half an inch space to intervene before completing the incision, thus allowing for the contraction of the urethra, as well as giving better shape to the much-prized but mutilated organ. After tying the arteries I made two stitches through the urethra on each side, attaching it to the corpus-spongiosum to prevent further contraction. I then made a few superficial stitches through the skin and corpora-cavernoso. After inserting a small glass tube into the urethra I washed the parts with a solution of peroxide of hydrogen (full strength), and directed a continuous moist application consisting of the following well-known medicines: bichloride of mercury, four grains; pure glycerin, four ounces; peroxide hydrogen, q. s. to make one pint of the lotion. This was applied continuously night and day by means of absorbent cotton for the space of eight days. I removed the stitches on the eighth day and found the wound healing very kindly. The patient having failed to keep the glass tube in the urethra necessitated my having to enlarge the opening, which I did on short notice with a pair of sharp-pointed scissors, preceded by a local application of a ten per cent. solution of cocain.

Immediately after cutting the urethra I made an application of a mild solution of perchloride of iron to the fresh edges of the incision, which kept them from healing, thus giving the patient a fairly good meatus urinarius.

This patient made a good recovery, and has remained entirely free from all recurrent symptoms of the disease, remarking, when I saw him last, that his greatest regret was he could not, as in former days, urinate against a perpendicular object.

CASE 3.—A lady, fifty-seven (57) years old, robust and apparently healthy; family history uncertain; mother died of pulmonary tuberculosis; one sister has tuberculosis and possibly carcinoma of the uterus. This patient complained of a lump in left breast, with considerable tenderness in the axillary space. On examination a small ulcer on the outside of the nipple and a large mass in the upper portion of the breast were readily discovered; also one or two excavations containing fluid or pus were found slightly protruding under the skin near the base of the nipple. There was no swelling in the axillary space nor of the cervical glands. Some tenderness extended above the breast in the direction of the axillary space.

With the efficient assistance of Dr. H. C. Beckett, of Scottsburg, who was the family physician of the patient, I removed the entire breast with the knife, making two carefully directed incisions from above downwards, afterwards carefully dissecting every trace of suspicious tissue. I then douched the entire wound with a solution of chloride of zinc (1–40) in warm sterilized water, after which I closed the wound with a continuous suture of aseptic silk, having previously adjusted a suitable drainage-tube. After making an application of a 5 per cent. solution of aristol and collodion on a small piece of iodoform gauze along the line of incision as an antiseptic cement, I proceeded to apply the usual dressing of iodoform gauze and carbolized absorbent cotton. This was followed by a carefully-applied bandage around the entire body. On the fourth day after the operation, I removed the drainage-tube, and on the eighth day the dressing and stitches, and was not surprised to find that union had taken place by first intention and everything in nice shape. This patient was operated upon August 14th, 1893; and I am happy to say she is a well woman to-day, having passed over the three-year limit safe and free from recurrent symptoms.

CASE 4.—Mrs. O., a lady sixty-two years old and in very feeble health. She had carcinoma of left breast which was caused, as she stated, by a severe blow from one of her grandchildren, five years previously. The family history of this patient is very unfavorable, her grandmother on her mother's side having died of cancer of the nose and face in her sixtieth year. One uncle and two aunts died of tuberculosis. One brother is now suffering from cancer of the head and neck. This lady was refused an operation by an eminent surgeon in Danville, Va., on account of her infirmity and general bad health.

Three months after this she applied to me for treatment. I advised an operation, preceded, however, by a preliminary course of constitutional treatment. Five weeks after this, assisted by Drs. Williamson and Faulkner, I amputated the entire breast with a good portion of the underlying tissue and muscle, which looked a little suspicious. I also cauterized the suspicious parts with a strong solution of chloride of zinc. Having adjusted a suitable drainage-tube, I closed the wound with a continuous suture of aseptic silk, and applied the usual dressing of antiseptic gauze and absorbent cotton. This patient made an uninterrupted recovery, notwithstanding her age and general infirmity. She was operated upon May 20, 1895, and up to this writing, she has enjoyed better health since the operation than for the previous five years.

Before reporting my next case, I desire to call attention to the important fact, that in operating for cancer of the breast, I invariably leave the axillary space uninvaded, notwithstanding the fact that the practice and teaching of some of our best modern surgeons on this subject are directly contrary to these views. If it be true that cancer partakes largely of the etiology of tuberculosis, as held by many able writers of the modern schools, it must necessarily be true that the organisms of cancer can be conveyed from one part of the human structure to any other part with as much ease and facility as that of tuberculosis. Hence we conclude that it would be worse than folly to enter the axillary space and deprive the body of important glands, at the same time knowing that the cancerous poison may be doing greater damage to other organs and infecting other parts of the human structure. Even admitting the common belief that cancer is conveyed only through the lymphatics, does it not seem unreasonable to remove the axillary glands in carcinoma of the breast, at the same time knowing that the other neighboring glands within the thorax and the great number of lymphatics in the neck and face may be infected with the same poison? I therefore conclude, if there is any virtue in removing one set of glands, there should also be the same indication for removing any or all of the glands of the body; for inasmuch as the lymphatics extend through the whole body and can convey a poison to any part thereof with about as much facility as to any special part or locality, I cannot think it is clear reasoning or good surgery to remove the axillary glands in the operation for carcinoma of the breast, and at the same time leave other infected glands in the body.

I therefore place myself on record as opposed to the common practice of removing the axillary glands in ordinary cases of carcinoma of the breast, unless we are fully convinced that the cancer is strictly limited to these special glands and the breast. It is just as much the duty of the surgeon to remove the inguinal or pelvic glands for carcinoma of the uterus, vulva, penis or scrotum. The same rule applies to the removal of the submaxillary and sublingual glands in epithelioma of the lip, nose, tongue and larynx.

CASE 5.—This lady was fifty-seven years old, the wife of a distinguished Lutheran minister and president of a noted college.

Her family history was favorable so far as I could ascertain. She was the mother of four children none of whom have ever shown any indications of the disease. This lady consulted me about the return of her menses and her profuse leucorrhea, as she termed them, not for one moment thinking that she was then suffering from any form of malignant disease. She was apparently in robust health, and had never suffered from any acute pain. In making a speculum examination, I readily discovered from the offensive discharge and its peculiar character, as well as from the external crater appearance of the cervix of the uterus, that this was a carcinoma of long standing. She informed me that she had suffered from repeated hemorrhages at various intervals for over eight months previous to consulting me. She also stated that she had suffered from a profuse watery, yellowish discharge from her womb for about five months. On examination, I found that this discharge contained lumps of putrid flesh varying in size from a split pea to a chestnut. These masses, on microscopic examination, proved to be cancer, at least the culture products correspond to those usually given and described in our text-books.

After two weeks' palliative treatment, consisting of curetting, and packing the uterus with antiseptic gauze, and the use of galvanic electricity, and not seeing any special improvement, I decided to give the patient the doubtful advantage of a more radical form of treatment. Accordingly, I wrote to her husband, who was in an adjoining State, to come at once with a view to accompany his wife to a first-class hospital. After a consultation with the husband and patient, we decided to go to Richmond to consult Dr. Hunter McGuire as to the propriety of subjecting the patient to a radical operation.

Dr. McGuire, who is always honest and frank toward his fellow-physicians, did not hesitate to express his opinion, after a thorough and careful examination, that the case was one of the very gravest, yet possibly an operable one. The husband, who, of course, was very anxious about his wife, after mature consideration, decided with me that we would go on to Baltimore and consult an eminent abdominal surgeon of that city. After a cursory examination, this eminent surgeon decided that the case was a favorable one for an operation, and advised complete extirpation of the uterus by the vaginal route. This gentleman's eminence as an abdominal surgeon, and the superior advantages of the world-renowned hospital with which he was connected, induced me to submit the whole matter to the patient and her husband, thus throwing the entire responsibility on their decision. They were not long in making up their minds to have the operation performed.

I shall forbear giving a description of the unfortunate operation that this eminent surgeon, with his staff of able assistants, performed, but suffice it to say, the patient died with a mutilated uterus intact and a severed ureter to tell the tale better than pen can describe it. The carcinoma which occupied the fundus and a large portion of the cervix, was left unmolested by the skilled (?) operator, notwithstanding the fact that this was, in the operator's opinion, a *most operable* case. While I would not detract from this eminent surgen's reputation as a gynecologist, yet I must frankly say he proved himself unpardonably deficient in surgical diagnosis, as well as in prognosis, in this one instance.

My past five years' experience in the treatment of this fell disease convinces me thoroughly that radical operations for carcinoma of the uterus, after twelve months' standing, is a travesty on modern surgery, and should be condemned by all honest surgeons and physicians who have at heart the real interest of our profession. I might go a step further, without transcending the limits of good logic, from a surgical point of view, and say, that *capital* operation for the treatment of any malignant disease that has passed the primary stage, should be abandoned and condemned.

Methinks I hear you ask, What shall we do with the inoperable cases, or those that have passed beyond the primary stage of the disease? In reply, I would say, first get your patient's mind composed and free from the horrible forebodings that usually accompany this dread disease, by building up the nervous system with nerve tonics, such as strychnia, phosphoric acid, and electricity. This should be followed at the proper time with reconstructive agents, such as iron, cod-liver oil and hypophosphites, etc.; bromide of arsenic, iodide of calcium internally, and carbid of calcium externally, have been extensively used in the treatment of this disease. Local treatment should be combined with the constitutional, and consists of hypodermic injections in and about the diseased structures of diluted alcohol and bichloride of mercury in the strength of one to five hundred (500) or one to one thousand (1,000), according to the type and malignancy of the disease. I have treated successfully a large number of cases in the primary stage with applications of caustic potash and Marsden's paste. In properly selected cases, this form of treatment, in my judgment, is superior to a bloody cutting operation with the knife, and I find that a patient will readily assent to this form of treatment, while he would otherwise reject the knife, and thus prolong the risk. Local anesthesia is a very important adjunct in the treatment, and should never be omitted when operating, either with the cautery or knife. I don't think a physician is ever justifiable, *under any conditions*, in telling a patient that he has a cancer. I have seen patients get apparently well by disabusing their minds of the disease without any treatment, except a placebo to divert the mind.

The day is not far distant when conservative surgery, aided by electricity, X-rays, and the many other modern agencies in use, will sound the death-knell of bold radical surgery.

In conclusion I would sound a note of warning to my fellow-practitioners against sending their malignant cases off to the bold, reckless, salaried hospital surgeon whose reputation is gauged by the number of radical operations performed and the number of females unsexed, instead of the actual number of cures made.

Far better for the patient, the family physician, and the medical profession at large to keep the suffering patient at home, even though his days on earth be numbered, than have him sacrificed in a world-renowned hospital at the hands of the reckless surgeon whose anxiety for notoriety is greater than his sense of right.

It is a sad picture, as well as a calamity to our profession, that so many of our fellow-mortals are to-day being sacrificed on the operating-table, whose lives might be prolonged months, and possibly years, under a rational and more conservative form of treatment.

REFERENCES.

Warren's Surgical Pathology and Therapeutics.
The International Annual, 1898.
Jour. Amer. Med. Assoc., July 9, 1898.

